# Medial femoral condyle free flap for carpo-metacarpal instability following hamate comminute fracture

**DOI:** 10.1007/s00402-022-04654-4

**Published:** 2022-10-19

**Authors:** Marco Borsetti, Luca Patanè, Silvia Germano, Enrico Cavalieri

**Affiliations:** 1 Division of Plastic Surgery, Hand Surgery and Microsurgery, Surgical Department of ASL Città di Torino, Maria Vittoria Hospital, Turin, Italy; 2 Department of Surgery “Pietro Valdoni” Plastic Surgery Unit, Sapienza University of Rome, Rome, Italy

**Keywords:** Hamate fracture, Medial Femoral Condyle flap, Osseous flap, Bone reconstruction, Hand trauma, Hand microsurgery

## Abstract

Complete reconstruction of the hamate bone has been reported in the literature mostly following cancer excision or avascular necrosis. For the exiguity of the tissue deficit, bone grafting has usually been used as treatment option for its rapidity and easiness to perform, even if a variable amount of bone resorption may occur. In traumatic cases, microbial contamination may jeopardize the success of a well performed bone graft and vascularised bone grafts may represent a better reconstructive option. Here we describe the first case reported in the literature of a patient underwent complete hamate reconstruction following trauma with an osseous medial femoral condyle free flap as vascularized arthrodesis between the capitate and the 4th MTC base, in order to stabilize the 4th and 5th finger and the ulnar carpo-metacarpal joint.

## Introduction

Preservation of congruent articular surfaces of the CMC joints is crucial to achieve satisfactory results following hamate fractures. If not correctly treated, the fracture may result in residual pain and swelling in the dorso-ulnar side of the carpus and reduction of hand grip strength up to 85%, usually developing a limited 4th and 5th finger active motion and other complications [1].

In the literature, the Medial Femoral Condyle flap has successfully been reported for the treatment of bone non-unions especially of the clavicle, humerus, elbow, forearm, wrist-scaphoid, lunate, phalanges, and metacarpals [2–5].

The current case report highlights the surgical technique and postoperative results for the treatment of a hamate comminute fracture with avulsion/extrusion of bone fragments with curettage and vascularized arthrodesis between capitate and the 4th MTC bone by mean of a cortico-cancellous vascularized medial femoral condyle (MFC) bone graft.

## Methods

### Presentation of case

**Fig. 1:**
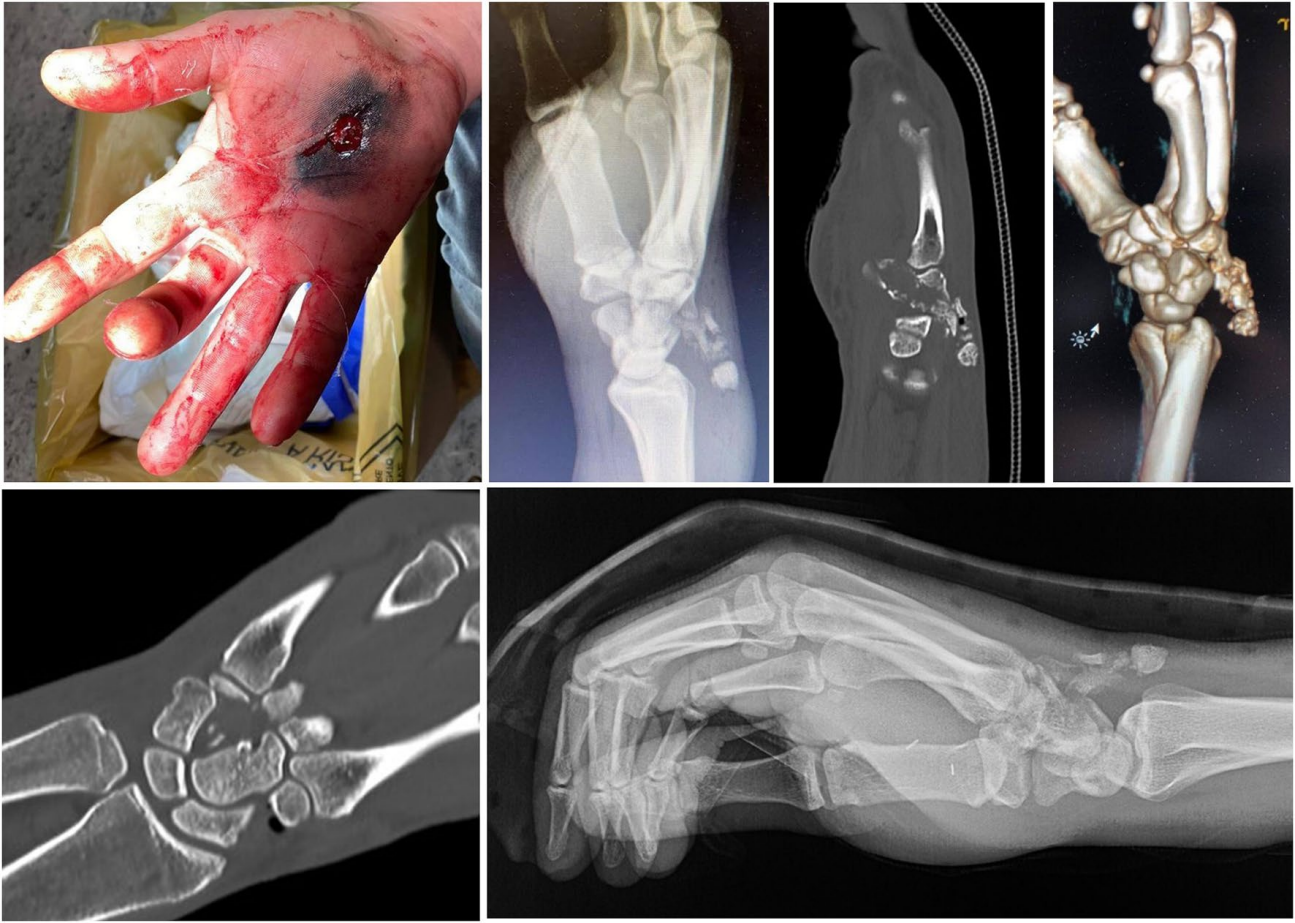
Penetrating injury of the right hand. X-ray scan and TC showed hamate bone comminute fracture and dislocation in the subcutaneous plane. After the first procedure in which debridement and nervous and tendinous reconstruction was performed, a plaster cast was applied

The patient was a 41-years-old right-handed man who sustained a penetrating injury of the right hand for a self-inflicted air-gunshot (Fig. 1). Physical examination revealed a deficit of the 4th and 5th fingers active mobility with flexion/extension ranges severely impaired. Severe pain was also reported by the patient. Clinically, tendinous and nervous continuity seemed not to be preserved. Wrist radiographs showed complete comminution of the hamate bone and revealed its dislocation in the subcutaneous plane. Favourably, no radial dislocation of the triquetrum and pisiform bones or proximal migration of CMC joints of the 4th and 5th metacarpals were observed. CT examination confirmed these findings.

Before surgery, treatment options were discussed with the patient considering the literature short-comings and lack of clarity regarding the possible surgical therapy and its outcomes. Other options examined were filling of the bony gap with antibiotic-loaded bone cement or bone grafting.

Due to the young age of the patient and his functionality needs, the Senior Author (M.B.) opted for a 2-stage approach implying a first surgical exploration with tendon/nerve repair and a second one with microvascular bone reconstruction and arthrodesis.

The patient underwent to a first operation in which debridement and exploration with a volar approach were performed. Rupture of the 4th and 5th finger deep (DDFT) and superficial (SDFT) digital flexor tendons was revealed and repaired by a 4-strand 3–0 suture of the DDFT. Additionally, a discontinuity of the motor branch of the ulnar nerve was detected and repaired following Guyon’s canal release. Bone fixation was not performed due to the high risk of infection. At the end of the operation, the hand was immobilized by a plaster cast (PIPJ at 120° and MFJ at 45°).

Following approximatively 30 days, once flexor tendon rehabilitation was completed, the second surgical step implied hamate bone reconstruction with the medial femoral condyle flap and its use as vascularized arthrodesis between capitate and the 4th metacarpal base.

### Surgical technique

In the second stage surgery, the patient was placed in the supine position with the leg flexed at both the knee and the hip in external rotation. The leg was elevated but not exsanguinated prior to tourniquet inflation to improve visualization of the vessels.

**Fig. 2:**
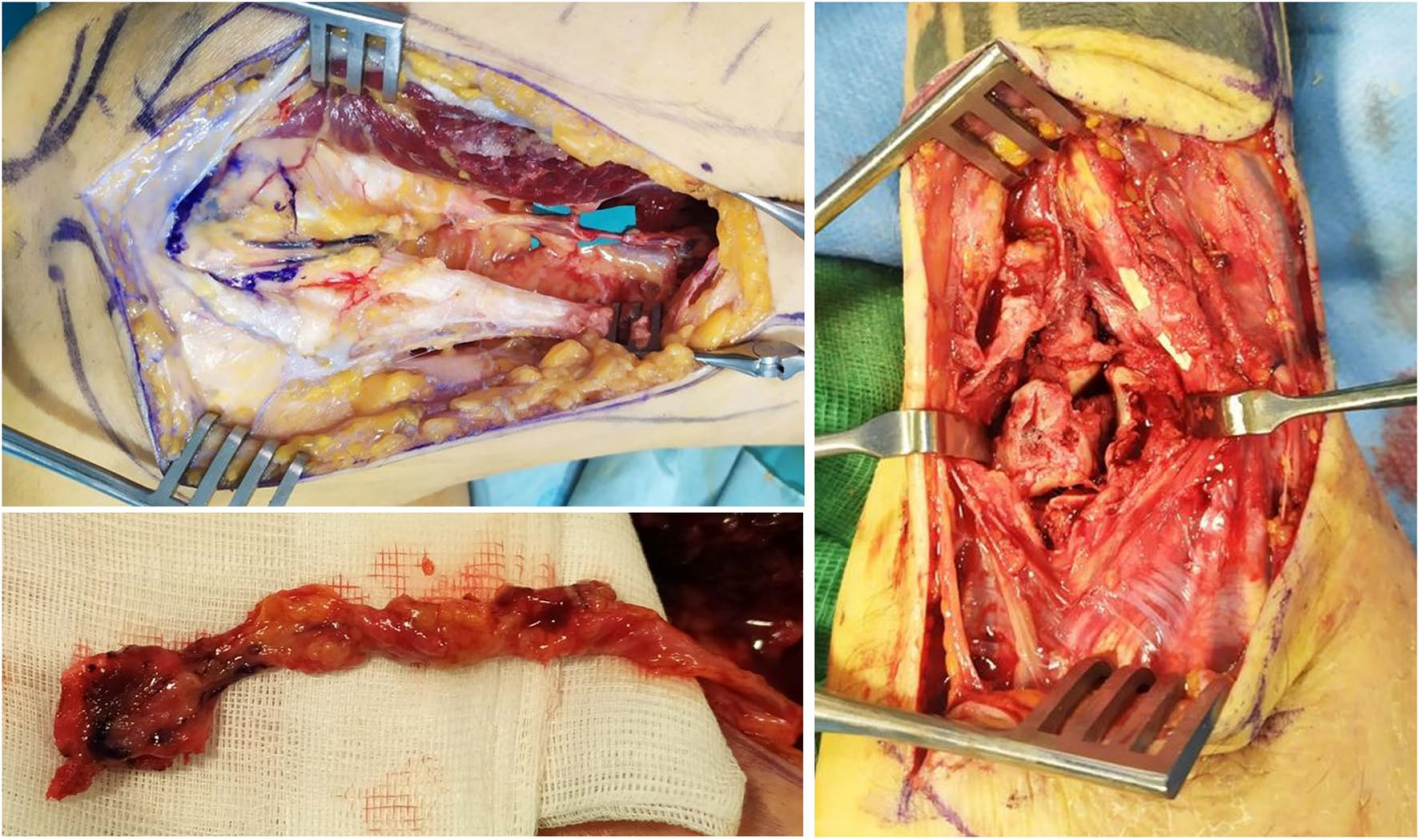
Descending Genicular Artery is exposed and dissected back to its origin. On the left, the entrance of the pedicle into the flap and the harvested Medial Femoral Condyle corticocancellous flap. On the right, inset of the flap through the dorsal incision

Under general anaesthesia, a mid-axial incision was made from the lower one third of the thigh over the adductor hiatus to the medial collateral ligament over the patella. Dissection was carried through the subcutaneous tissue using electrocautery. The fascia of the vastus medialis was incised and the muscle retracted anteriorly to expose the descending genicular vessels at their distal part, which are intimately connected with the periosteum. At this level, afferent branches of the artery to the bone were identified. Retrograde dissection was then performed over the DGA back to its origin at the adductor canal (Fig. 2). Subsequently, the second arterial source of the flap (Supero-Medial Genicular Artery) was dissected proximally back to its origin at the popliteal artery.

Once arteries were isolated, the condyle was cleared of all superficial soft tissue and the required flap was drawn on the condyle of the femur, avoiding to extend over the articular surface and the medial collateral ligament. The periosteum was then cut with cautery and the cortex elevated with a chisel.

A 1.5 × 1.5 cm Cortico-cancellous bone flap was elevated on the Supero-medial genicular artery, which presented adequate vascular caliber. The tourniquet was then released to perform accurate haemostasis, the donor site was closed in layers with absorbable suture and a drain was placed in the thigh.

The recipient site was prepared simultaneously with the flap harvest following tourniquet inflation. A transverse bayonet approach to the dorsal wrist was performed, preserving the sensitive branch of the radial nerve and the superficial venous system. In the subcutaneous plane, multiple avulsed hamate fragments were noted and excised. The midcarpal joint was accessed through the hamate and its residual parts articulating with the capitate and the 4th and 5th metacarpals were excised. Intraoperatively, the midcarpal joint and the 4th and 5th metacarpal bases were considered unstable as they tended to collapse.

The receiving radial vessels were prepared through an incision in the anatomical snuffbox. The flap was inset and hold in place by a 1.2 mm K-wire inserted in the 3^d^ metacarpal base.

Microsurgical arterial anastomosis was performed end-to-end to the dorsal carpal branch of the radial artery using 10–0 nylon interrupted sutures, away from the surgical site to avoid local edema and inflammatory response. One comitant vein was then similarly anastomosed end-to-end to a collateral branch of the radial vein with 9–0 nylon interrupted sutures. Adequate flap bleeding and positive patency test confirmed flap vascularization.

To protect the anastomotic site, a 1.4 mm K-wire was then used to stabilize the radio-carpal joint. The wounds were closed and a volar plaster cast was placed.

## Results

**Fig. 3:**
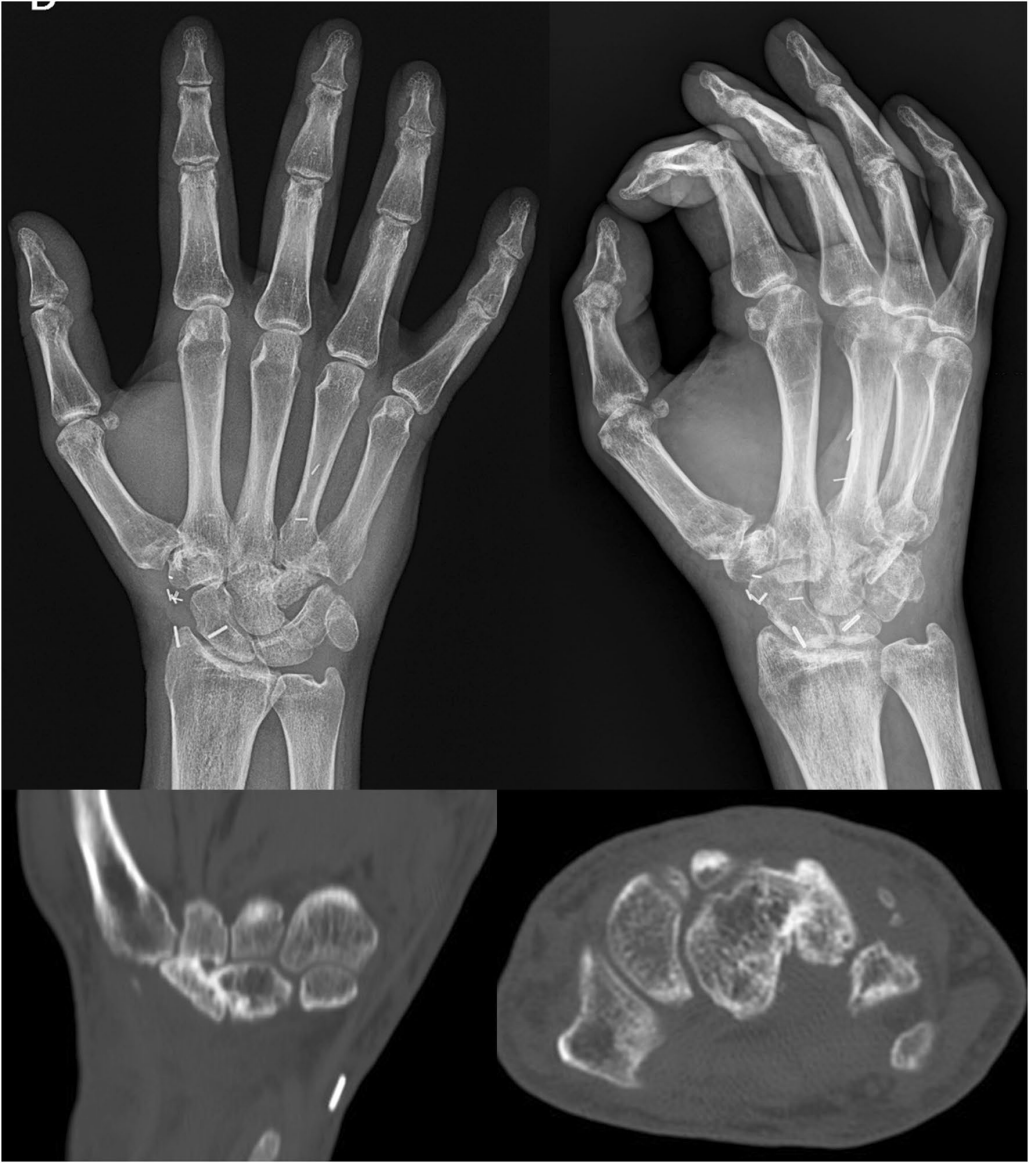
X-ray and CT scan, 16 weeks and 6 months postoperatively. Flap viability and ulnar carpal-metacarpal joint stability are evaluated

**Fig. 4:**
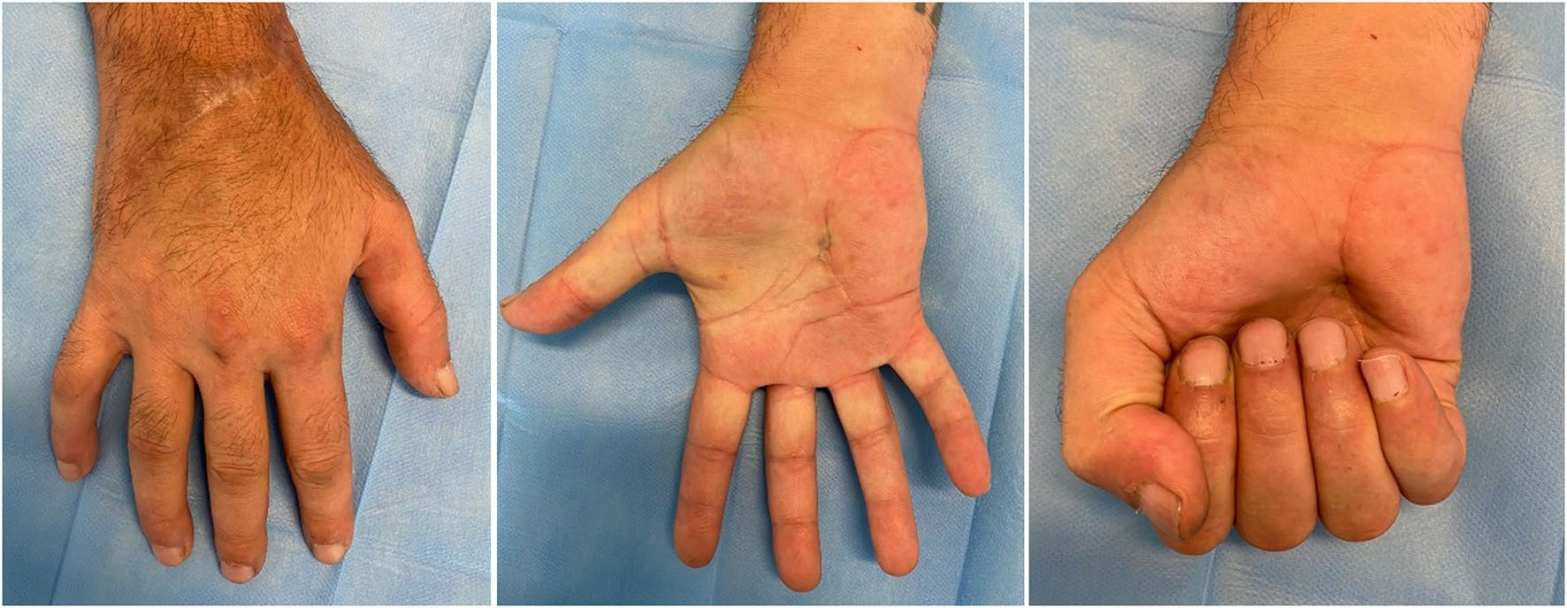
6 months follow-up, active flexion/extension ranges of movement are restored

Amoxicillin + clavulanic acid (1 g /8 h, for 5 days) and pain-relief medications were administered until wound healing was reached. The patient was encouraged to ambulate the following day and discharged from the hospital 3 days after surgery. On the recipient site, postoperative immobilization was achieved with a pinstripe shower that was applied avoiding compression over the site of the anastomoses. Rehabilitation started 7 days postoperatively with passive-active digit motion. After 4 weeks of immobilization, the patient transitioned to a removable brace for further 2 weeks. Postoperative radiographs were obtained at 16 weeks and a CT scan was obtained at 6 months, to verify healing (Fig. 3). The evaluation revealed good bone vitality and no collapse of the 4th metacarpal base due to arthrodesis stability. K-wires were removed at 4 weeks postoperatively. At 6 months follow-up (Fig. 4), the patient gained complete ROM during flexion and extension of the finger, with no residual ulnar sided pain and instability or impingement on the triquetrum.

## Discussion

When complete reconstruction of the hamate bone following open trauma is needed, in our opinion autologous vascularised bone graft should be highly considered for their low resorption rates. Moreover, non-vascularised bone grafts are more susceptible to infection that, in a trauma-related contaminated field, may be enhanced with this practice. For this reason, we opted not to utilize external material, such as K-wires or surgical screws, in the first reconstructive stage to lower the risk of infection. In the literature, autogenous non-vascularised iliac crest bone grafts and vascularised radius bone grafts [6] were used for hamate complete reconstruction following oncological resection [7], avascular necrosis [8–12] and haematogenous osteomyelitis [17] but, in some reports, reconstruction was not considered necessary [8, 10, 13–16]. In this case, the instability of the 4th and 5th finger constrained to perform a vascularized arthrodesis for a successful outcome and avoiding complications derived from the hamate fracture and restoring an adequate carpal/metacarpal ratio. Consequently, the choice of performing a vascularised flap was considered as the best one, aiming to utilize its good vascularity to carry vital tissues in a devitalized and contaminated environment.

## Conclusion

Treatment of hamate fracture/dislocation depends on the characteristics of the trauma and on the reparability of the remaining bone fragments. In this case, the use of the medial condyle free flap was useful to reconstruct the hamate bone and restore a normal carpal/metacarpal ratio, permitting to perform a vascularised arthrodesis to treat the residual instability of the 4th and 5th digits and the ulnar carpo-metacarpal joint.

## Declarations

### Research involves human or animal participants

All procedures followed were in accordance with the ethical standards of the responsible committee on human experimentation (institutional and national) and with the Helsinki Declaration of 1975, as revised in 2008.
